# Usage of EMBRACE^TM^ in Gujarat, India: Survey of Paediatricians

**DOI:** 10.1155/2014/415301

**Published:** 2014-10-30

**Authors:** Somashekhar Nimbalkar, Harshil Patel, Ashish Dongara, Dipen V. Patel, Satvik Bansal

**Affiliations:** ^1^Department of Paediatrics, Pramukhswami Medical College, Anand, Karamsad, Gujarat 388325, India; ^2^Central Research Services, Charutar Arogya Mandal, Anand, Karamsad, Gujarat 388325, India

## Abstract

*Aim*. EMBRACE^TM^ is an innovative, low cost infant warmer for use in neonates. It contains phase change material, which stays at constant temperature for 6 hours. We surveyed paediatricians using EMBRACE^TM^ regarding benefits, risks, and setup in which it was used in Gujarat. *Methods*. Questionnaire was administered telephonically to 52 out of 53 paediatricians. *Results*. EMBRACE^TM^ was used for an average of 8.27 (range of 3–18, SD = 3.84) months by paediatricians. All used it for thermoregulation during transfers, for average (SD) duration of 42 (0.64) m per transfer, 62.7% used it at mother's side for average (SD) 11.06 (7.89) h per day, and 3.9% prescribed it at home. It was used in low birth weight neonates only by 56.9% while 43.1% used it for all neonates. While hyperthermia was not reported, 5.9% felt that EMBRACE^TM^ did not prevent hypothermia. About 54.9% felt that they could not monitor the newborn during EMBRACE^TM^ use. Of paediatricians who practiced kangaroo mother care (KMC), 7.7% have limited/stopped/decreased the practice of KMC and substituted it with EMBRACE^TM^. *Conclusions*. EMBRACE^TM^ was acceptable to most but concerns related to monitoring neonates and disinfection remained. Most paediatricians felt that it did not hamper KMC practice.

## 1. Introduction

Lack of thermal protection is one of the major challenges faced by developing nations for newborn survival [1]. In India, the prevalence of hypothermia varies widely but recent estimates in normal newborns in community settings are around 31% and about 32% in hospital settings, but these included mostly normal weight newborns [2, 3]. The prevalence can be estimated to be even higher for low birth weight newborns. A greater proportion of child deaths in the western and southern parts of India are attributable to low birth weight and premature babies [4]. Almost 2.8 million neonatal deaths occurred in the year 2013 globally, of which 73% deaths occurred during the first seven days of life [5]. Neonatal mortality contributes to more than half of the under-five mortality in countries such as India [5]. The rates of decline in the last decade have been the slowest for neonatal mortality [6]. Every 1°C below 36°C on admission increased the odds of late onset sepsis by 11% and of death by 28% [7].

Hypothermia in newborns is rarely a direct cause of death but rather exists as a comorbid condition along with birth asphyxia, neonatal infections, and preterm birth and leads to a substantial amount of mortality. The hypothalamus along with various endocrine organs is responsible for the process of thermoregulation in newborns. In LBW and preterm babies, these mechanisms are overwhelmed resulting in metabolic disturbances which ultimately result in neonatal death, either directly by hypothermia or indirectly [1]. In preterm infants there can be a rapid drop in temperature by almost 0.5°C to 1°C per minute. Cold ambient temperature, intrahospital transfers, low temperature of hospital beds and poor warm chain practices during resuscitation, and late onset of breastfeeding, and so forth, are some additional factors which hasten the onset of hypothermia [8]. In a recent study of 300 consecutive neonates, almost 47% neonates had hypothermia in spite of about 75% being institutional deliveries [9]. In developing countries neonatal hypothermia often goes unnoticed and unaddressed [10].

Various measures to prevent hypothermia are practiced by healthcare providers and these vary with the population of neonates being catered to. Maintaining a delivery room temperature of more than 25°C, wrapping the baby in clean linen, drying, using baby hat, starting skin-to-skin contact with mother after delivery also termed as kangaroo mother care, placing the neonate under radiant warmer, delaying bathing, usage of chemical mattresses, and wrapping the neonate in plastic are all practiced by healthcare providers [11]. Kangaroo mother care has shown to prevent hypothermia along with other benefits to the child which include increased breast feeding and better weight gain [12]. An estimated 4,50,000 babies can be saved worldwide every year if supportive care in the form of kangaroo mother care is provided by healthcare providers [13]. The most recent systematic review on kangaroo mother care in low birth weight infants has shown a reduction in the risk of mortality (risk ratio of 0.60, 95% confidence interval (CI) 0.39 to 0.92) as well as reduction in the risk of hypothermia (risk ratio of 0.34, 95% CI 0.17 to 0.67) and reduction of nosocomial infection/sepsis (risk ratio of 0.45, 95% CI 0.27 to 0.76) when evaluated at discharge or 40-41 weeks postmenstrual age. Even at the latest follow-up the KMC was associated with reduction of mortality and sepsis [14]. In areas where there are fewer healthcare providers and even lesser resources a simple technique to prevent and manage hypothermia is required. The EMBRACE^TM^ warmer is a small sleeping bag-like apparatus which has in its back a reusable pouch of phase change material that can be heated to 37°C and can maintain that temperature for several hours. It is cheap, reusable, portable, and hygienic and does not require constant electricity [15]. This has been developed by students at Stanford University and has been marketed in India for the last two years. There have been no published trials of EMBRACE^TM^ though there are four studies which are listed on clinical trial registries [16–19]. We surveyed users of EMBRACE^TM^ in Gujarat for their assessment of its benefits and the various settings in which it was being used.

## 2. Materials and Methods

The survey questionnaire was developed and was consensually validated by four neonatologists at the neonatal intensive care unit (NICU) of Shree Krishna Hospital, Karamsad (Appendix). Over a span of 2 months, the questionnaire was administered by telephone to 52 out of a population of 53 practitioners who were using EMBRACE^TM^ at their setups in different cities across the state of Gujarat. One paediatrician declined to answer the survey. The study was approved by the institutional ethics committee.

*Instrument*. The questionnaire comprised of 22 questions regarding the use of EMBRACE^TM^, for instance, what were the indications for using EMBRACE^TM^, the average duration of the use of the product per day, major advantages and disadvantages as experienced by the paediatricians while using EMBRACE^TM^, and the impact of EMBRACE^TM^ on the practice of kangaroo mother care. Demographic information of the hospitals at which the participating paediatricians practiced was also taken into account.

## 3. Results

The paediatricians have been using EMBRACE^TM^ since a mean time of 8.27 months (range = 3–18 months, SD = 3.84). The product is mainly used for thermoregulation during transfers (100%) and during hospital stay when the baby is at the mother's side (62.7%) and is even prescribed at home in a small proportion of cases (3.9%). When used for transfers, EMBRACE^TM^ was utilized for a mean (SD) duration of 42 (0.644) minutes per transfer, and when used to keep the baby warm while at the mother's side, it was used for a mean duration of 11.06 h (SD = 7.98) a day.

We attempted to determine the target population for EMBRACE^TM^ use. We found that 56.9% of paediatricians used it for low birth weight neonates and 43.1% paediatricians used it for normal weight and low birth weight neonates. None of the paediatricians used the product after delivery unless the neonate was vitally stable. Of all the paediatricians, 54.9% used EMBRACE^TM^ within 12 h of delivery, 31.4% used it within 12–48 h after delivery, and 13.7% used it after 48 h of delivery.

We determined that 98% of the paediatricians were comfortable using the product routinely and 100% of the paediatricians felt that the babies were comfortable while placed in EMBRACE^TM^. All of the paediatricians (100%) found EMBRACE^TM^ to be a useful product for transfers, both intra- and interhospital. More than half of the paediatricians (58.8%) did not report any disadvantage or problems while using EMBRACE^TM^, while the rest encountered one problem or the other. Of the paediatricians, 14.3% felt that EMBRACE^TM^ was too costly and not worth its price, 33.3% reported issues related to heating and temperature regulation related issues, 33.3% paediatricians had issues related to cleaning, disinfection, size issues (too large for ELBW infants), charging, and monitoring, and 19% paediatricians found a combination of the above-mentioned issues.

Of the paediatricians using EMBRACE^TM^ for babies kept at the mother's side, 94.1% did not notice any weight gain in infants and 62.7% found it to prevent hypothermia when the baby was at the mother's side. But, 5.9% paediatricians did not find EMBRACE^TM^ to be a useful product to prevent hypothermia in newborns. No doctor reported hyperthermia as a consequence of EMBRACE^TM^ use.

Out of all the paediatricians using EMBRACE^TM^, 54.9% felt that they could not monitor the newborn effectively when placed within EMBRACE^TM^. Of those who felt that they could monitor the infant, 95.7% monitored only pulse oximetry, whereas 4.3% said that they monitored both the chest rise of the baby and pulse oximetry readings.

All the participating paediatricians (100%) had heard about kangaroo mother care, out of which 98% were formally trained in kangaroo mother care (KMC), and 74.5% paediatricians practiced KMC at their setup. Of the paediatricians who practiced KMC, 7.7% have limited/stopped/decreased the practice of KMC and substituted it with EMBRACE^TM^ for thermoregulation. All 7.7% of these doctors were involved in private sector. On further questioning, healthcare providers gave various reasons for reducing KMC. These included “more compliance of EMBRACE^TM^ with nurses and relatives,” “counselling for KMC requires 30 minutes,” “training staff is a headache,” “hygiene issues in mother,” “EMBRACE^TM^ is equivalent to KMC,” “KMC is not possible in private setups,” and “there is no space to give KMC.” 90.4% paediatricians included in the study were from private sector whereas only 9.6% were from public sector. Among the doctors who were involved in private practice, 27.6% did not practice KMC at all whereas all the doctors in public sector practiced KMC at their setups. EMBRACE^TM^ was being used for babies kept by mother's side by 38.2% private practitioners, whereas only 1 out of the 5 practitioners in public sector used the product for this purpose. Ninety-two percent paediatricians levied no charges on the patients for using this product, whereas 8% paediatricians levied nominal charges for maintenance of the product. No doctor charged more than 500 rupees per day.

## 4. Discussion

The current survey reports on the usage of EMBRACE^TM^ of almost all healthcare providers having EMBRACE^TM^ in Gujarat in mid-2013. In a recent survey on neonatal resuscitation carried out in Gujarat there were a similar number of participants that participated and hence we believe that this survey on EMBRACE^TM^ is representative of current neonatal practice in Gujarat [20].

Although published literature on the benefits of EMBRACE^TM^ is not available the fact that it has a relatively large number of users within a short period of time is an indicator of the need for a low-cost product to prevent neonatal hypothermia. It can be argued that KMC is a better and proven intervention than EMBRACE^TM^ for management of hypothermia. However providing KMC encompasses support from the family and the healthcare providers. Though the current study has not addressed this aspect, the authors surmise that this support is not available due to rapid uptake of EMBRACE^TM^ and a sizeable number of providers stopping KMC. Additionally though a neonate can be transferred while in KMC position with mother/relative, it is often not feasible because of unavailability of mother/relative to provide KMC during transfers or in case of urgent intra- or interhospital transfers of neonates when there is not enough time to prepare the mother/relative for providing KMC, and so the use of a low-cost product like EMBRACE^TM^ to prevent neonatal hypothermia is warranted.

The results indicate that these healthcare workers are comfortable with using EMBRACE^TM^ suggesting that the design of the product is simplistic in nature and does not require extensive training. Incubators and radiant warmers are complex devices which are costly and require training and their usage practices vary with different neonatal units, experience, and knowledge [21]. Availability of EMBRACE^TM^ allows healthcare providers to use it in situations wherein they would have utilized a radiant warmer with all of its attendant technical requirements, especially when KMC would be nonfeasible to implement as already mentioned. Using EMBRACE^TM^ as a sleeping bag kind of mattress allows the paediatricians to reduce the complexity of care. Usage of EMBRACE^TM^ for transport had universal appeal as compared to kangaroo mother care which requires training and extensive support before it can be effectively used. However the short duration of transport does not shed any information of value, as increased neonatal mortality occurs when the transport duration exceeds 1 h [9].

Healthcare providers shared their concerns about cleaning, disinfection, and temperature regulation of the neonates. This is surprising as the training of EMBRACE^TM^ and the literature pack provided describes these in detail. The concerns related to the EMBRACE^TM^ warmer being large for ELBW babies are notable. EMBRACE^TM^ is provided with a cushion for smaller neonates. However there was no specific problem related to usage in ELBW babies. Almost 40% healthcare providers expressed their apprehension about the effectiveness of the product in preventing hypothermia although they were all comfortable using it. Whether it is a design issue or a training issue cannot be commented upon, due to the design of our survey. More detailed understanding of these concepts is needed. There was not a single report of hyperthermia which possibly indicates the safety of EMBRACE^TM^ though our study design was not designed to study safety features. This is significant as EMBRACE^TM^ is being utilized in community settings across the world with minimal supervision by healthcare personnel. In a recent study of a thermal regulation bundle there was 2% incidence of temperatures >38°C in a very low birth weight (VLBW) population [22]. Inability to monitor the baby was another lacuna that EMBRACE^TM^ has in a hospital setting. EMBRACE^TM^ has an internal monitor for temperature of the phase change material but baby's temperature cannot be monitored. The healthcare providers also monitored oxygen saturation in these babies.

Kangaroo mother care is being proposed as a better alternative to conventional care with wide ranging benefits including reduction of neonatal mortality, hypothermia, and length of hospital stay [23]. There are many more benefits that accrue from KMC such as reducing maternal postpartum depression, neonatal pain reduction, prolonged breastfeeding, and positive infant development and parent child bonding. This has led to the concept that premies are exterogestational foetuses that require provision of continuous skin-to-skin contact to promote maturation [24]. It is well acknowledged that only a fraction of the neonates requiring KMC are receiving it. It probably stems from viewing KMC as an option available to poor people in low income countries, from absence of knowledge or from lack of engagement of parents [24]. Measures to accelerate KMC need to be put in place [25]. It is in this context that the finding that almost 75% of the surveyed healthcare providers were using KMC was gratifying. However, almost 8% had reduced/stopped the practice of KMC after beginning to use EMBRACE^TM^. While EMBRACE^TM^ does prevent hypothermia it does not have the additional advantages that KMC has. The authors believe that this finding has serious implications for neonatal health in the long term. Efforts to promote KMC will possibly face stiff resistance from healthcare providers using EMBRACE^TM^ as it is a therapy which is easy to administer and even easier to monitor. KMC on the other hand requires training of staff, counselling of parents who are often unwilling and if given an alternative may choose EMBRACE^TM^, monitoring, and larger space utilization in the hospital. The benefits of KMC are more in the long term and are not really seen or felt by the healthcare provider. With the introduction of technology in the form of ultrasound machines in the 1980s there has been a reduction of the sex ratio in India to alarming levels [26]. There has been no head-to-head trial of EMBRACE^TM^ versus KMC. It may be unethical to conduct one in most settings as equipoise is no longer present. Yet the authors believe that a trial which compares these modalities of neonatal thermal management is necessary, albeit in a design which is ethical. EMBRACE^TM^ may yet be utilized as an adjunct to KMC in situations where provision of KMC may be challenging. Studies focusing on the situations where these challenges can be faced and complimented by EMBRACE^TM^ need to be undertaken.

## 5. Conclusions

EMBRACE^TM^ is being used not only for transport but also for stabilization of the neonate in the neonatal intensive care unit. Paediatricians are using it in both LBW and normal weight newborns and some have concerns related to disinfection and monitoring of neonates. However, most paediatricians felt that it did not hamper the practice of kangaroo mother care. As EMBRACE^TM^ usage increases, researchers working in this area need to focus on developing strategies to improve the uptake of kangaroo care while providing evidence based guidelines for the usage of EMBRACE^TM^. Efforts need to be made to publish studies related to efficacy and effectiveness of EMBRACE^TM^ in clinical care of neonates.

## Appendix

(1) Name.
(2) Since when has ${\text{EMBRACE}}^{\text{TM}}$ been used? (in months).
(3) In what kind of babies did you use ${\text{EMBRACE}}^{\text{TM}}$? Normal birth weight, low birth weight, or both.
(4) What was the purpose of use? Purpose of use—transfers, prevent or treat hypothermia when baby is out of NICU or is with mother, both.
(5) Were there any charges levied if any? (in rupees).
(6) Within how much time did the use of ${\text{EMBRACE}}^{\text{TM}}$ start after delivery? Immediately, after initial stabilization (within 6 hours), within 6–48 hours, or after 48 hours.
(7) What was the duration of use of ${\text{EMBRACE}}^{\text{TM}}$ per day in newborns for transfers? (in hours).
(8) What was the duration of use of ${\text{EMBRACE}}^{\text{TM}}$ per day in newborns for purposes other than transfers? (in hours).
(9) Did you notice any pattern of weight gain in babies after the use of ${\text{EMBRACE}}^{\text{TM}}$? Yes, No.
(10) Are you comfortable in using ${\text{EMBRACE}}^{\text{TM}}$ or not? Yes, No.
(11) What are the major benefits of ${\text{EMBRACE}}^{\text{TM}}$ noticed? No benefits, transfers, maintaining warm temperatures while at mother’s side, or combination of any.
(12) Have any newborns developed hypothermia while placed in ${\text{EMBRACE}}^{\text{TM}}$? Yes, No.
(13) Have any newborns developed hyperthermia while placed in ${\text{EMBRACE}}^{\text{TM}}$? Yes, No.
(14) Are newborns comfortable while placed in ${\text{EMBRACE}}^{\text{TM}}$? Yes, No.
(15) Do you use ${\text{EMBRACE}}^{\text{TM}}$ for transfers? Yes, No.
(16) Are you able to monitor newborns kept in ${\text{EMBRACE}}^{\text{TM}}$? Yes, No.
If yes, what parameters can you monitor? Respiration (chest rise), pulse oximetry, or both.
(17) Is there any disadvantage of using ${\text{EMBRACE}}^{\text{TM}}$? Yes, No.
If yes, costly product, heating and temperature regulation related issues, other technical issues like size too big for ELBW babies, charging, cleaning, and monitoring issues, or combination of any.
(18) Have you heard of kangaroo mother care? Yes, No.
(19) Have you been trained in kangaroo mother care formally or otherwise? Yes, No.
(20) Have you ever used kangaroo mother care at your setup before today? Yes, No.
(21) Did you stop/limit/decrease using KMC after buying and using ${\text{EMBRACE}}^{\text{TM}}$? Yes, No, or not applicable as never used KMC.
(22) What is the number of hours that you subject the baby to KMC? (in hours).
(23) Address of the hospital.
